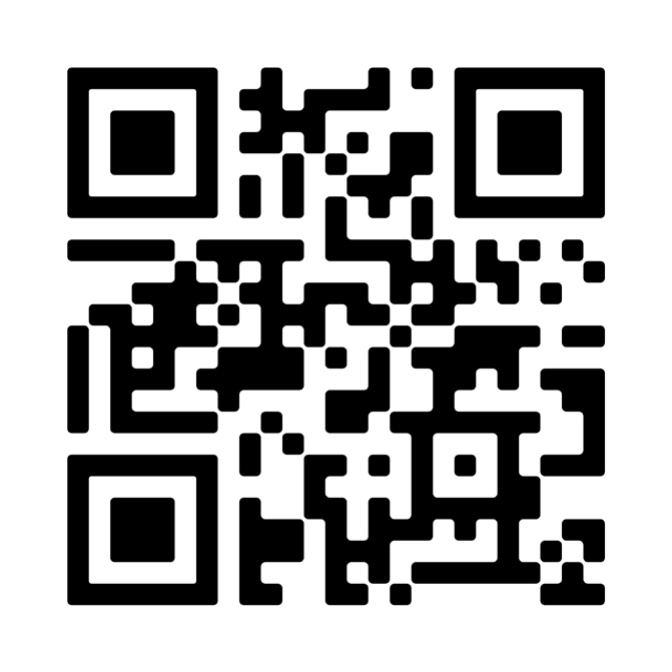# Laparoscopic transabdominal cerclage in a pregnant woman after fertility-sparing treatment for early-stage cervical cancer: an operative technique in ten steps

**DOI:** 10.52054/FVVO.16.2.018

**Published:** 2024-06-28

**Authors:** M Pavone, N Bizzarri, M Goglia, L Lecointre, A Fagotti, G Scambia, D Querleu, C Akladios

**Affiliations:** Institute of Image-Guided Surgery, IHU Strasbourg, France; UOC Ginecologia Oncologica, Dipartimento di Scienze per la Salute della Donna e del Bambino e della Sanità Pubblica, Fondazione Policlinico Universitario A. Gemelli, IRCCS, Rome, Italy; Research Institute Against Digestive Cancer, IRCAD Strasbourg, France; Department of Medical and Surgical Sciences and Translational Medicine, Faculty of Medicine and Psycology, Sapienza University of Rome, Italy; Department of Gynecologic Surgery, Hôpitaux Universitaires de Strasbourg, France; ICube, UMR 7357 CNRS, University of Strasbourg, France

**Keywords:** transabdominal laparoscopic cerclage, fertility sparing, cervical cancer, pre-term birth

## Abstract

**Introduction:**

Fertility-sparing treatments are increasingly used in patients with early-stage cervical cancer. The residual shortened cervix might increase the risk of preterm birth. When a vaginal cerclage is not technically feasible, a laparoscopic transabdominal cerclage (LAC) could be offered before or after conception. In this article, we show how to safely perform a post-conceptional LAC in patients with insufficient residual cervical length for vaginal cerclage.

**Methods:**

A 34-year-old patient in the twelfth week of gestation who previously underwent repeated conisation for cervical cancer FIGO stage IA1 in 2021 was referred for cervical stenosis, which required a subsequent vaginal tracheoplasty. She became pregnant 3 months later. Ultrasound monitoring of the cervix showed a 15 mm cervical length. A step-by-step LAC in a pregnant woman was performed.

**Results:**

The Doppler velocimetry of the uterine arteries at the end of the procedure was normal. No intraoperative or postoperative complications were reported. The estimated blood loss was 100 mL and the total operative time of 120 min. The patient was discharged on the third postoperative day. A caesarean section was performed at 36 weeks of gestation for spontaneous contractions with excellent obstetric (male, 2860 gr) and neonatal outcomes.

**Conclusion:**

LAC in pregnancy, although made more difficult due to the size of the uterus, is a safe and feasible procedure combining the advantages of minimally invasive surgery with excellent obstetric result.

## Learning objective

This video shows how to perform a post- conceptional transabdominal laparoscopic cerclage in a young woman with no sufficient cervical length for a vaginal approach.

## Introduction

In developed nations, approximately 8.6% of live births involve premature delivery (Blencowe et al., 2013; WHO Global report, 2023) that accounts for 35% of all neonatal deaths, representing one of the leading causes of neonatal mortality and morbidity (Liu et al., 2010). One contributing factor to preterm birth is cervical insufficiency, which affects 1% of all pregnancies and is responsible for 8% of recurrent miscarriages (Drakeley et al., 1998; McDonald, 1980). Cervical insufficiency is characterised by painless dilation of the cervix without uterine contractions, resulting in second- trimester births in an otherwise normal pregnancy (Frega et al., 2018). Moreover, ultrasound scans often reveal a shortened cervical length, which is frequently indicative of early-stage cervical insufficiency (Roman et al., 2016; Han et al., 2020). The placement of a cervical cerclage can effectively treat cervical insufficiency by providing support to the cervix. This intervention, when indicated, can lead to an extended gestation period and a decrease in preterm births, as well as a reduction in neonatal mortality and morbidity (Roman et al., 2016). In recent years fertility-sparing treatments have increasingly been developed in patients with early- stage cervical cancer (Gabriele et al., 2019; Fanfani et al., 2021). At the same time, a short or scarred residual cervix after conservative surgery such as conisation or trachelectomy appears to increase the risk of second-trimester loss and preterm birth (Frega et al., 2018; Alvarez et al., 2018).

When cervical insufficiency is identified during the initial pregnancy, a cerclage can be performed based on ultrasound indications, or if there is evidence of cervical dilatation (Shennan et al., 2022). Shirodkar (1955) was the first who reported a transvaginal cerclage (TVC) lately modified by McDonald (1980), however, when this approach is not technically feasible due to a short or scarred cervix, transabdominal cerclage (TAC) (Benson and Durfee, 1965) could be offered via laparotomy or minimally invasive surgery (MIS) (Guan et al., 2023; Moawad et al., 2018; Shennan et al., 2020; Montaguti et al., 2023).

**Figure 1:**
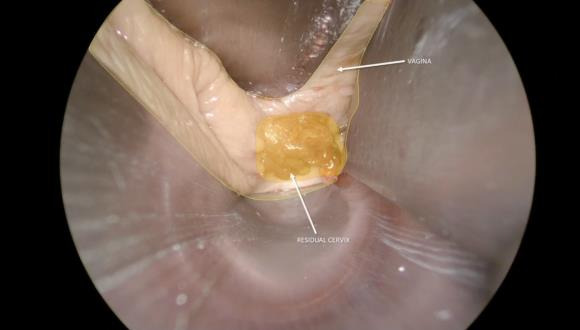
Residual Cervix after tracheloplasty.

The advantages of MIS for patients over the laparotomic approach are clear (Johnson et al., 2005) and a recent meta-analysis also found improved obstetric outcomes when choosing laparoscopy (Moawad et al., 2018). Laparoscopic cerclage can be performed before or after conception even if the post-conceptional approach is limited by gestational age (GA). The size of the gravid uterus, the need to minimise movement to avoid complications, and the increased risk of bleeding make the procedure more complex in this time frame (Moawad et al., 2018). The aim of this article is to show the technical procedure to perform safely a post-conceptional LAC in patients who have undergone fertility- sparing surgery for early-stage cervical cancer with insufficient residual cervix length for vaginal cerclage (Figure 1).

### Case presentation

We report the case of a 34-year-old patient in the twelfth week of gestation who previously underwent repeated conisation for FIGO stage IA1 cervical cancer in 2021. She was referred for cervical stenosis, which required a subsequent vaginal tracheloplasty with the surgical excision of the fibrotic cervical tract in 2022. She became pregnant 3 months later. Ultrasound monitoring of the cervix showed a 15 mm cervical length. The study was conducted in accordance with the Declaration of Helsinki, and formal approval from the Ethical Committee/ Institutional Review Board was not required for video articles’ publication. However, written informed consent for treatment and the analysis of data for scientific purposes was obtained from the patient.

### Surgical procedure

**Figure 2:**
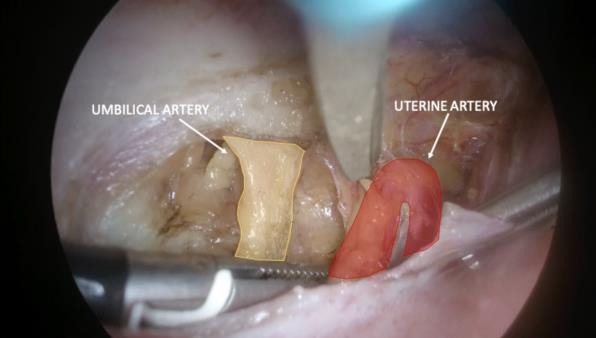
Left para-vesical space dissection. The umbilical artery and uterine artery are shown.

**Figure 3:**
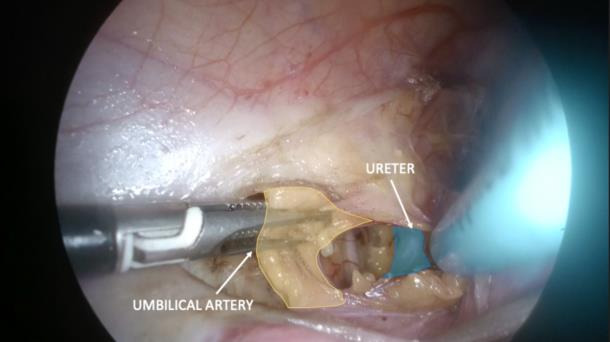
Left para-vesical space dissection. The umbilical artery and ureter are shown.

**Figure 4:**
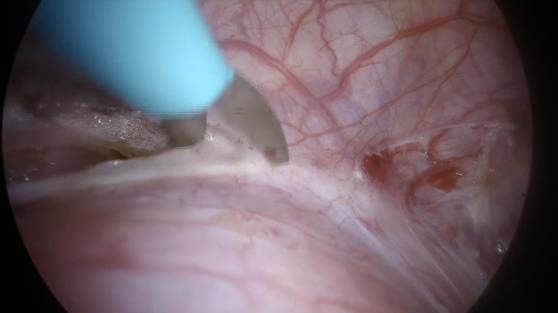
Vesical-uterine fold dissection.

**Figure 5:**
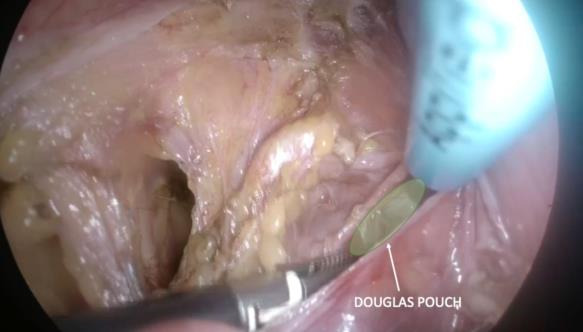
Windowing of the broad ligament. Anterior access to the Douglas Pouch is shown.

**Figure 6:**
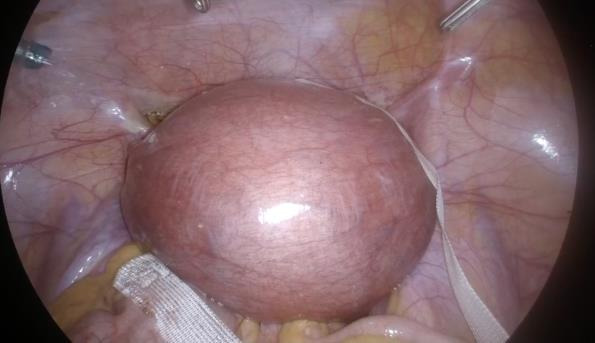
3 mm monofilament polypropylene sling (Aris®, Coloplast).

**Figure 7:**
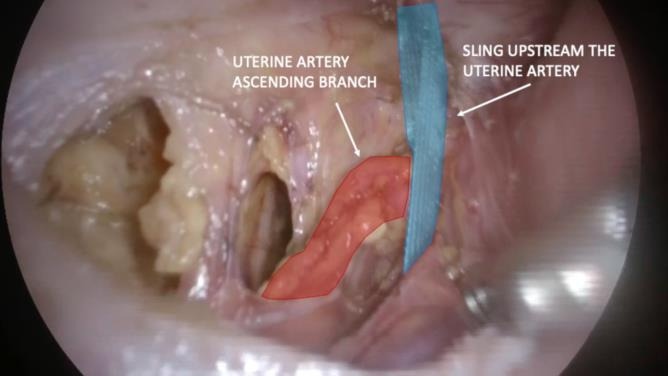
Sling introduction upstream of the vessels. Sling and uterine arteries are shown.

**Figure 8:**
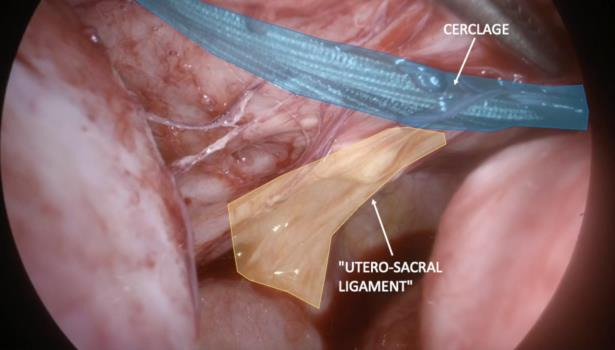
Posterior vision of the positioned cerclage. Cerclage and uterosacral ligament are shown.

**Figure 9:**
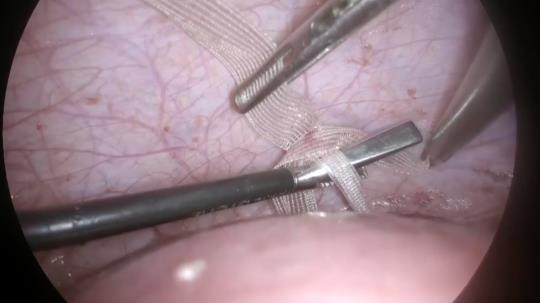
Cerclage anterior knot.

Under general anaesthesia the patient was in a dorsal lithotomy position with both legs supported in Allen stirrups and arms alongside the body. Step 1: Pneumoperitoneum was obtained with Veress needle up to 13–15 mm Hg of CO2. The 12-mm optic trocar was placed above the umbilicus. The patient was placed in the Trendelenburg position. Two lateral and one mid-line laparoscopic 5 mm trocars were introduced under vision. Step 2: After systematic exploration of the pelvis, the procedure began. Step 3: Dissection of the para-vesical spaces was performed bilaterally to expose the ureters and the branches of the uterine artery (Figures 2 and 3). Step 4: The anterior peritoneal fold was dissected up to the reunification with uterine vessels and the bladder was then reflected (Figure 4). Step 5: A window was created through the broad ligaments in the avascular space medially to the uterine vessels. Step 6: Anterior access to the pouch of Douglas was gained (Figure 5). Step 7: A 3 mm monofilament polypropylene sling (Aris®, Coloplast) adapted for the procedure was introduced in the abdominal cavity (Figure 6). Step 8:The tape was thus positioned from posterior to anterior through the broad ligament windows on each side upstream of the uterine arteries and around the isthmic portion of the uterus (Figure 7). Step 9: The posterior cerclage right position above the “utero- sacral ligaments” was checked (Figure 8). Step 10: The sling was loosely tied anteriorly with five intracorporeal knots (Figure 9).

## Results

The Doppler velocimetry of the uterine arteries at the end of the procedure was normal. The following day, a uterine artery Doppler confirmed normal flow bilaterally. No intraoperative or postoperative complications were reported. The estimated blood loss was 100 mL and the total operative time of 120 min. The patient was discharged on the third post-operative day. A caesarean section was performed at 36 weeks of gestation for spontaneous contractions with excellent obstetric (male, 2860 gr) and neonatal outcomes. There was no need for the cerclage removal during the caesarean section.

## Discussion

This article reports the operative technique demonstration of a post-conceptional laparoscopic transabdominal cerclage. The procedure, performed by an expert gynaecological surgeon, was carried out without complications and with excellent obstetric outcomes. Conservative management for pre-invasive lesions of the cervix, as for early-stage cervical cancer, is increasing due to the efforts in fertility sparing management of gynaecological diseases (Fanfani et al., 2021; Morice et al., 2022). When the cervix is shortened or scarred, cervical incompetency is more frequent, and a vaginal approach for cerclage positioning may not be feasible (Gomes-Da-Silveira et al., 2015). Benson and Durfee (1965) were the first who introduced the abdominal cerclage, making this option available also for patients who are not possible candidates for the vaginal approach. To date, no randomised trials are available for the comparison of the laparoscopic and laparotomic approaches. Systematic reviews on retrospective series suggest the absence of differences in obstetric outcomes, survival, and gestational age at delivery, but a disadvantage in terms of hospital stays, aesthetic results, and complications when laparotomy is chosen (Hulshoff et al., 2020). Among the advantages of a TAC approach is the possibility of being performed under local-regional anaesthesia (spinal). Besides the shorter execution time, the reduced anaesthetic impact can play a crucial role, especially when the cerclage is positioned post-conception. The robotic approach is also a viable and safe procedure, as proposed by Guan et al. (2023), even if indications are still not clear and the cost is higher if compared with laparoscopy. Even if LAC can be offered pre or post-conception, in the latter, surgery can be more challenging. The increased size of the pregnant uterus makes difficult the instruments’ movements in the reduced space. Moreover, to limit the possible fetal complications and risk of bleeding, it is crucial not to exceed in the uterine manipulation. The trocars insertion can be dangerous if the pregnancy is advanced with an augmented risk for complications also linked to the increased vascularisation of the organ at this time (Ades et al., 2019). The complication rate is higher than 19% if the LAC is performed during the pregnancy (Whittle et al., 2009). Therefore, to reduce possible complications, it is recommended to perform LAC prior to pregnancy or within the first trimester of pregnancy. In the presented case, the traditional laparoscopic cerclage technique is shown, which involves, after vascular dissection, the placement of a tape medially to the ascending branch of the uterine arteries. Choosing a needle-free technique helps in reducing surgical gestures, uterine manipulation, and possible complications. Modified and simplified variants of the presented technique include the fenestration of round ligaments without vessel skeletonisation with a lateral approach to uterine vessels. This procedure can be adopted when cases are particularly complex, to minimise the risks of bleeding even if it may result in a reduction of blood supply to the uterus due to the closure tying the tape of the corporal branches of the uterine arteries (Zhao et al., 2022). However, as for trachelectomies, where uterine vessels are cut, the blood supply that comes from the ovarian pedunculus is enough to maintain the foetal growth during the pregnancy with no anomalies described in the literature in the umbilical Doppler velocimetry after this approach (Abdel Azim et al., 2021). In addition to the effectiveness of the knot, the fibrotic peritoneal reaction around the sling helps in the reduction of cervical incompetence. Some authors suggest peritonealising the sling anteriorly and posteriorly to increase its compressive effect (Smith et al., 2017). The knot can be closed anteriorly or posteriorly to the uterine isthmus without significant differences (Clark and Einarsson, 2020). Posterior closure may be useful in reducing the possibility of adhesions and make the subsequent caesarean section less complex.

When possible, the application of a prophylactic cerclage after both vaginal and laparoscopic trachelectomy should be considered avoiding a secondary surgery (Gomes-Da-Silveira et al., 2015; Pavone et al., 2023). New techniques have been proposed to overcome the limits of the previous approaches: a retroperitoneal isthmic cerclage via vaginal natural orifice transluminal endoscopic surgery (vNOTES) was recently suggested to simplify the vaginal technique (Baekelandt, 2023), however studies on its feasibility are still ongoing.

The preferred intervention for women who have experienced unsuccessful vaginal cerclage is transabdominal cerclage. In comparison to low vaginal cerclage, transabdominal cerclage demonstrates superiority in lowering the risk of early preterm birth and fetal loss among women with a history of unsuccessful vaginal cerclage. This advantage is not observed with high vaginal cerclage (Shennan et al., 2020). The cerclage can be left in position permanently and caesarean sections are routinely scheduled for these patients. If complications occur, however, a laparoscopic removal can be offered as reported by Agdi and Tulandi (2008) in the case of a 19-week gestational patient with oligoamnios. Customising surgery based on the patient’s characteristics and the complexity of the case is, however, advantageous. By following the precautions described above, complications can be avoided, and the LAC performed safely.

## Conclusions

Laparoscopic transabdominal cerclage in pregnant women with a shortened cervix after conservative surgical procedures, although made more difficult due to the size of the uterus and the need to minimise its mobilisation, is a safe and feasible procedure in expert hands combining the advantages of minimally invasive surgery with excellent obstetric result.

## Video scan (read QR)

https://vimeo.com/904379559/9b8788669b

**Figure:**